# Evaluation of an improved picket fence style test for routine MLC positional QA

**DOI:** 10.1002/acm2.14567

**Published:** 2024-11-29

**Authors:** Michael Barnes, Therese Standen, Renee Blackmore, Peter Greer

**Affiliations:** ^1^ Department of Radiation Oncology Calvary Mater Hospital Newcastle Newcastle New South Wales Australia; ^2^ School of Information and Physical Sciences University of Newcastle Newcastle New South Wales Australia

**Keywords:** EPID, MLC, picket fence, quality assurance (QA), 87.55.Qr

## Abstract

**Purpose:**

The aim was to develop and evaluate an EPID‐based MLC positional test that addresses known weaknesses of the picket fence test and has sufficient accuracy so that the AAPM MPPG 8.b. MLC position action limit of ± 0.5 mm can be implemented.

**Methods:**

Weaknesses and inaccuracies in the picket fence test were identified and a new test plan and analysis algorithm named stakitt was developed. Stakitt was evaluated for repeatability and for sensitivity on the Varian TrueBeam linac with both Millennium MLC and HDMLC and on a Varian Clinac with Millennium MLC. Sensitivity was tested via deliberate introduction of errors into the test plan of magnitude: ± 0.1, ± 0.5, ± 1.0 and ± 1.5 mm. Measured sensitivity was compared to published sensitivity of the picket fence test. Additionally, a use case was presented based on results from a TrueBeam linac to highlight the effect of MLC backlash on MLC positions at non‐zero gantry angles.

**Results:**

Repeatability was observed to within 0.04 mm (3 SD) with the TrueBeams being more repeatable than the Clinac. The deliberately introduced errors were accurately measured to within 0.28 mm and were comparable to the traditional picket fence. Reduced accuracy was recorded for the HDMLC small leaves, which was attributed to an apparent variation in measured leaf width across the range of travel, which impacted the measurement of the leaf tip position. The clinical use case demonstrated variability in MLC leaf positions between gantry 90° and gantry 270° that were of the magnitude of the MLC backlash.

**Conclusion:**

The stakitt test addresses the weaknesses of the picket fence test and has accuracy appropriate for implementation of a ± 0.5 mm action limit. However, such an action limit may not be currently practical at non‐zero gantry angles due to the impact of MLC leaf backlash.

## 1 INTRODUCTION

Modern radiotherapy treatment techniques, such as intensity‐modulated radiotherapy (IMRT) and volumetric modulated arc therapy (VMAT), utilize multileaf collimators (MLCs) to create intra‐beam varying beam apertures to deliver complex beam fluences as determined by the treatment planning system (TPS) to optimize the dose distribution in the patient. It is widely accepted that accurate MLC leaf positions are required for accurate delivery of both IMRT^1^, ^2^, ^3^, ^4^, ^5^, ^6^, ^7^, ^8^, ^9^ and VMAT^10^, ^11^ treatment types. With the advent of stereotactic type treatment techniques, the clinical importance of MLC leaf positional accuracy is considered greater, as the impact from inaccuracies is magnified due to the small aperture sizes involved and the need for higher spatial accuracy of dose gradients given the hypo‐fractionated treatment schedules.^12^, ^13^ Hence, positional accuracy quality assurance (QA) of the MLC is paramount.^14^, ^15^, ^16^, ^17^ The most recent AAPM guidance on MLC positional QA is from Medical Physics Practice Group (MPPG) 8.b,^17^ which recommends that the previously recommended weekly qualitative MLC positional test be replaced with a quantitative test with action limit tightened to ± 0.5 mm. MPPG 8.b. also notes that in general “the frequency and rigor of MLC QA tests must increase.”

The “picket fence” test (or “garden fence” test in some literature) is a widely used technique to verify MLC leaf positions as part of a routine MLC QA program.^2^, ^18^ The picket fence test was originally developed using radiographic film^19^, ^20^ but has since been translated onto the linac electronic portal imaging device (EPID) for measurement convenience.^21^, ^22^, ^23^, ^24^, ^25^, ^26^, ^27^ The picket fence test consists of irradiation of MLC patterns designed to create narrow strips, or “pickets,” either by a series of fields a few centimeters wide that slightly overlap (standardly by 1 mm) or narrow bands of, standardly, 1 mm width evenly spaced. From an integrated image of the MLC pattern, the pickets for each leaf pair are traditionally assessed against all of the others. The picket fence test was originally designed for qualitative assessment but has since been quantified.^21^, ^22^, ^28^, ^29^

Quantification of the picket fence test is problematic due to a number of weaknesses associated with both the test pattern and its analysis. The known weaknesses of the picket fence test that have been addressed and improved upon in this study include the following.

1. In the picket fence test, each picket is formed by the positioning of opposed MLC leaves. If the opposed leaves have positional error in the same direction, then the position of the picket will be shifted. If the opposed leaves are in error in opposite directions, then the picket width will be varied. Although not always done, these effects require that both picket position and width both be analyzed to then be able to determine which individual MLC leaf's position is in error. It would be preferable to have a test that allows measurement of positional accuracy of each of the opposed leaves to allow easy assessment of which leaf is in error.
2. The 1 mm width of the pickets are subject to small‐field dosimetry considerations due to the picket consisting of the overlap of penumbrae from opposing leaves. This was intentional in the original film‐based qualitative picket fence as the narrow pickets exhibit high visual sensitivity to sub‐millimeter changes in leaf pair gap width. However, it also means the full width at half max (FWHM), normally used for quantifying picket gap width, is non‐linear with physical gap width due to reduced central axis (CAX) maximum dose compared to that in broad beam geometry.^30^ Hence, a calibration procedure is necessary, along with mathematical curve fitting, in order to accurately quantify measured gap results. Such a calibration procedure is problematic as without a ground truth MLC gap deliveries with intentional errors are required for which the magnitude of the introduced errors is assumed. This assumption can be minimized but not completely removed by repeating the calibration procedure multiple times across multiple machines. However, it would be preferable to have a test that did not require a calibration procedure to measure gap widths, but was determined from first principles.
3. With the traditional picket fence test picket position and gap are often measured relative to the other intra‐picket leaves. Thus, the method is insensitive to systematic positional errors across the whole bank, such as a mis‐calibration. Instead, spatially referencing to beam CAX, as has been done previously,^22^ offers a means of quantifying leaf positions in absolute terms, which subsequently allows comparison of measured positions with respect to expected leaf positions from the planned delivery.
4. For both the Varian Millennium and HD MLC's, the leaves travel independently within a carriage, but the carriage itself can also shift, mid‐treatment to increase the available field size for the large field IMRT technique. Such shifts are known as carriage shifts and during the large‐field‐IMRT technique the beam is held whilst the carriage and leaves move into the next position. The correct shift of the carriage and the positioning of the leaves post shift are required for accurate MLC positioning, but are often not included in traditional picket fence test plans.
5. Finally, most EPID‐based quantitative picket fence tests do not account for image rotations such as those caused by collimator and EPID rotation, which have the potential to result in small unaccounted for measurement errors. Robustness of the measurement would be improved by accounting for these rotations.

The aim of this study was to develop and evaluate an improved picket fence style test that addressed these identified weaknesses in the standard picket fence test and to provide a test method that could meet the requirements of AAPM MPPG 8.b. recommendations for routine MLC positioning QA. The newly developed test is locally known as the “Stakitt” test, which means “picket” test in Norwegian in reference to the nationality of one of the authors.

## 2 METHODS

### 2.1 Materials

The stakitt test pattern delivery DICOM plan file was created in MATLAB (MathWorks Inc., Massachusetts, version R2019a). The plan file was then imported into the Eclipse treatment planning system (TPS) (Varian Medical Systems, version 15.6.05) for delivery in clinical mode. The stakitt plan was delivered on two different Varian linac models (Clinac 2100Ix and TrueBeam) including two types of MLCs (Millenium120 and HDMLC). aS1000 (Clinac) and aS1200 (TrueBeam) EPID panel types were used to capture the delivery. The methodology should theoretically be translatable to any standard linac, MLC and EPID panel type, although this has not been tested.

### 2.2 Stakitt test

#### 2.2.1 Test pattern

**FIGURE 1:**
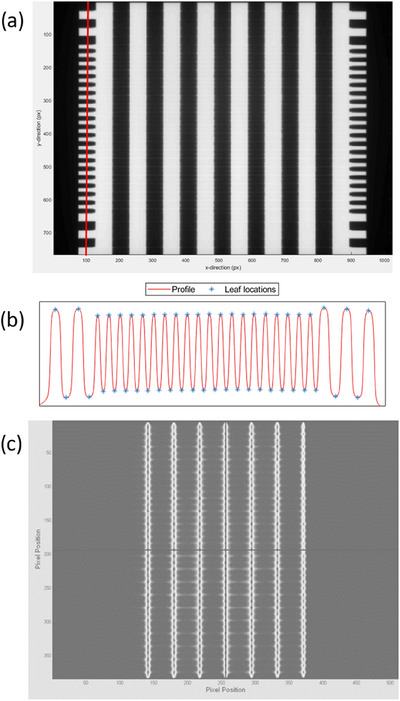
a) Example stakitt delivery EPID image showing the 6 central 2 cm spaced stakitts and the comb patterns on either side of the image used to locate individual leaves. The vertical red line represents where the profile is taken through the left side comb pattern to detect the individual leaves. b) Example profile through the comb pattern along the red line of (a) showing the peaks and troughs used to locate the centers of leaves. c) Example picket fence test EPID image for comparison to a) to highlight the differences in the picket fence and stakitt deliveries.

The stakitt test pattern consists of six evenly distributed 2 cm wide MLC‐defined fields (termed stakitts) with 4 cm center‐to‐center spacing between each (Figure 1a). This aspect of the method is adapted from Bayouth et al.^31^ The 2 cm width removes the small field dosimetry considerations of the standard picket fence and means that individual leaf positions can be measured as the maximum signal in the field does not vary significantly with small changes in field width, which significantly complicates the analysis. The outermost MLC patterns on both sides of the delivery are in the form of a comb pattern created by alternating protruding and retracted leaves. This comb pattern is used to locate individual leaves in the image to allow measurement along the line of trajectory. The test pattern is delivered in air to the EPID in integrated acquisition mode at 100 cm source‐detector distance (SDD) at the four cardinal gantry angles using the step‐and‐shoot technique with a 6 MV beam delivering 480 monitor units (MUs). An MLC bank carriage shift occurs between the two central stakitts.

#### 2.2.2 Beam central axis determination

The center of collimator rotation is used as the spatial reference point. This allows for leaf positions to be measured in absolute rather than relative to the other leaves in the stakitt as is common with the traditional picket fence test. To find the sub‐pixel point on the EPID panel which corresponds to the beam central axis (CAX), two jaw‐defined 10 cm x 10 cm fields are delivered to the EPID at collimator angles 90° (C90) and 270° (C270). The two images are acquired at source to detector distance (SDD) 100 cm in integrated acquisition mode at gantry angle 0° (G0). Beam center in each image is located as the midpoint between the 50% penumbrae in both crossplane and inplane profiles, which have been interpolated to provide sub‐pixel precision, taken through the center of the image. The CAX position is defined as the average of the beam centers between C90 and C270 images. This point is used as the spatial reference point for all subsequent measurements. Due to EPID panel positioning reproducibility, the EPID panel is not moved between CAX and Stakitt image acquisition.

#### 2.2.3 Mechanical sag correction

The EPID coordinates corresponding to the CAX are gantry angle dependent due to mechanical sagging of the EPID panel with gravity.^32^ The CAX position at non‐zero cardinal gantry angles is adjusted for EPID and gantry sag based on a linac‐specific measured EPID sag map according to the method of Rowshanfarzad et al.^32^ Delivery is with the field defined by a circular stereotactic cone, which is largely sag free compared to MLC's or jaws, whilst the gantry rotates through a full 360° arc. The sag map is determined via cine‐EPID imaging at 7.5 frames per second with 6 frame averaging. The sub‐pixel EPID coordinates that correspond to the center of the cone constitute the CAX at that specific gantry angle. The results are normalized relative to G0 CAX position. Medium‐term stability of the sag map has been demonstrated^32^ so the relative sag map is stored and applied to the measurement session G0 CAX value to correct the CAX for the other cardinal gantry angles. The sag map is checked independently of the stakitt measurement as part of the departmental monthly QA program.

#### 2.2.4 Analysis algorithm

On the stakitt delivery image, the algorithm locates individual leaves using the peaks and troughs of an interpolated line profile through the comb patterns on either side of the image, see Figure 1a. Each leaf trajectory is then determined as the line between corresponding leaves on either side. This corrects for any rotations between the collimator and the EPID panel. Along the leaf trajectories, leaf tip positions are determined by interpolation of the 50% radiation penumbrae and corrected for EPID radiation field offset (RFO), which accounts for differences in positions defined by the light field edge (used in DICOM) and the 50% radiation field edge.^33^ The 50% point is determined from local maximum and minimum values in the profile along the leaf travel. The 2 cm spacing between stakitts was chosen to be large enough to that these maxima and minim would be relatively stable in magnitude. Leaf positions are determined for each individual leaf from both MLC banks in all six stakitts for leaves visible on the image. Absolute leaf positions are then calculated as the distance of the measured leaf position from the measured CAX. The measured leaf positions are compared against the nominal distances from the plan.

The stakitt algorithm graphically outputs deviations from nominal leaf positions for each leaf in each MLC bank in a histogram format. Results from opposing leaves are also combined to inform upon variation in the MLC gap to highlight cases where opposing MLC leaves deviate from nominal in different directions and hence may be of clinical significance. Positive deviations indicate leaves are retracted and hence gap widths are larger than expected, whereas negative deviations indicate leaves are extended and gap widths are smaller than expected. Out of tolerance results are displayed in a separate figure and table form. A summary of the identified picket fence weaknesses and the improvements introduced into stakitt to address them are presented in Table 1.

**TABLE 1:** Summary of the identified picket fence test weaknesses and how they have been addressed in the Stakitt methodology.

Picket fence weaknesses		Addressed in Stakitt	
1	Leaf pair analysis only makes it difficult to diagnose which leaf is in error	✓	Individual leaf plus leaf gap analysis makes clear which leaf is in error while still allowing assessment of the clinically meaningful leaf gap parameter.
2	Gap width requires calibration	✓	Gap measured directly, no calibration required
3	Leaf pair analysis relative to other leaves	✓	Leaf positions measured in absolute referenced to measured collimator rotation axis (CAX)
4	Carriage shifts not assessed	✓	Carriage shift impact on leaf position accuracy assessed
5	Effect of collimator or EPID panel rotations not accounted for	✓	Collimator and EPID rotations accounted for via measurement along the leaf trajectories located in the image.

### 2.3 Evaluation of the method

#### 2.3.1 Stakitt algorithm consistency

The consistency of the algorithm was demonstrated by running the algorithm five times on a single test image.

#### 2.3.2 MLC repeatability

Short‐term MLC repeatability was assessed by performing five consecutive stakitt tests at G0 and calculating the standard deviations (SD) in measured leaf positions within each stakitt. These were averaged and the repeatability taken as three standard deviations (3 SD). MLC repeatability was determined for three combinations of linac and MLC types; Truebeam STx fitted with an HDMLC, Truebeam fitted with a Millennium120 MLC, and Clinac fitted with a Millennium120 MLC.

#### 2.3.3 Sensitivity analysis

The algorithm was evaluated by delivering the stakitt test pattern with deliberate MLC positional errors introduced into the plan and comparing the measured deviation to expected relative to the plan with no errors and assessed in the context of the repeatability results. Measurements were performed on the same three combinations of linac and MLC types as testing of MLC repeatability. The MLCs were initialized prior to plan deliveries and all deliveries were done at G0. Errors in leaf positions and gap widths were simulated with error magnitudes chosen near but within the action limit, at action limit, and outside the action limit. The in‐house action limit at the time used for both leaf position and gap width was 1 mm.^14^ Leaf positional errors were introduced in five non‐adjacent individual leaves of magnitudes ± 0.1, ± 0.5, ± 1.0, and ± 1.5 mm for the inner leaves (HDMLC: 2.5 mm leaves, Millennium120: 5 mm leaves) and ± 1.0 mm for the outer leaves (HDMLC: 5 mm leaves, Millennium120: 10 mm leaves). This mimics random leaf positioning errors. Gap width errors were simulated by shifting opposing leaf pairs of five adjacent leaves, which mimics systematic leaf positioning errors. Magnitudes were ± 0.1, ± 0.5, ± 1.0, and ± 1.5 mm for the inner leaves and +1.0 mm for the outer leaves where positive values indicate larger gap width relative to expected widths. The magnitudes were chosen such that no single leaf exceeded the positional action limit of 1 mm. Sensitivity of the method was then assessed by comparing measured leaf position and gap width deviations (from expected) in the error‐free delivery to the measured deviations in the error‐induced delivery. The measured sensitivity of stakitt was compared to results of the department's previous in‐house EPID‐based picket fence test as presented in Rowshanfarzad et al.^22^

### 2.4 Clinical use case

**FIGURE 2:**
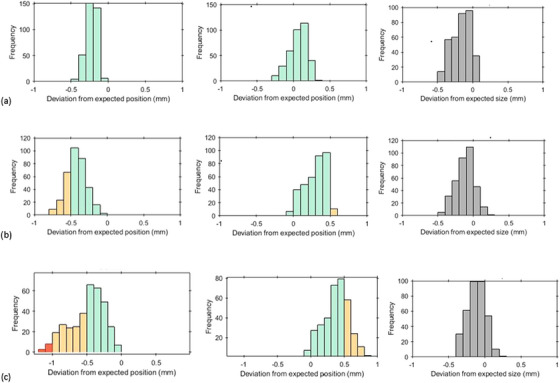
Stakitt clinical use case results. Left column provides Bank A deviations from expected, center column provides Bank B deviations from expected and right column presents measured gap deviations from expected. Row A is for G90, Row B is for G0, and Row C is for G270. Positive values indicate retraction away from the gap (gap expansion) and negative values indicate extensions into the gap (gap reduction).

During monthly clinical use of the stakitt test, it was observed on a TrueBeam STX linac equipped with HDMLC that there was a consistent offset in the measured leaf position error histograms between measurements at G90 and G270 for both MLC banks (Figure 2).

**FIGURE 3:**
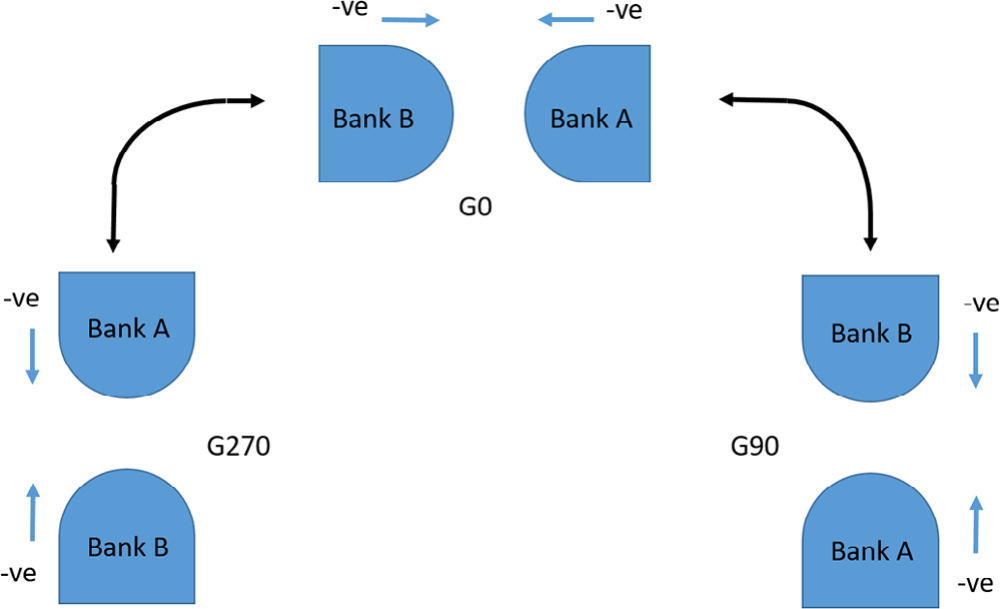
Schematic showing the positions of each MLC bank at different gantry angles from the viewpoint of looking towards the linac from the patient couch. The coordinate system of negative directionality indicating an extension of the leaf (minimizing of the gap) is also shown. (‐ve = negative).

At G90, MLC Bank B is above Bank A. As such, gravity is working to extend the leaves out from Bank B and retract them in Bank A. The situation is reversed at G270. See Figure 3. In Figure 2, the histogram for Bank A at G90 shows a slight negative offset becoming even more negative at G270. However, the histograms for Bank B show a slight positive offset at G90 becoming even more positive at G270. The result is that the gap remains approximately constant between G90 and G270. It was hypothesized that the observed shift for individual banks between gantry angles could be explained by mechanical play in the MLC leaf/carriage system (backlash) and the resulting gravitational impact on leaf position at the cardinal gantry angles when gravity is acting in the direction of leaf travel. The hypothesis was tested via performing the vendor‐supplied MLC backlash test and reconciling the results with the observed stakitt data.

#### 2.4.1 Backlash investigation

Stakitt images were acquired at G270, G0, and G90 in clockwise order using a collimator angle of 0°. Data acquisition was repeated three times and the results averaged. The in‐built Varian MLC leaf and carriage backlash test accessible through Service Mode (Truebeam V2.7 MR4) was used to investigate the backlash values for each MLC leaf and both carriages. During this test, each leaf is slowly driven into an infrared beam. When the leaf breaks the beam, it is stopped and is then retracted. The difference in motor counts between the leaf breaking the beam and the leaf leaving the path of the beam corresponds to a distance. This distance is the inherent backlash of a given leaf. The test also provides a backlash value for both carriages. The MLC leaf and carriage backlash test was performed five times at the Varian recommended G0. Backlash values for all leaves on each bank were averaged and compared to the mean stakitt results for each MLC bank. This process was repeated at G90 and G270.

## 3 RESULTS

### 3.1 Evaluation of the method

#### 3.1.1 Stakitt algorithm consistency

The stakitt test algorithm was found to be consistent with no differences observed to 4 decimal places when running the algorithm five times on the same test image.

#### 3.1.2 MLC repeatability

The MLC repeatability was 0.02  and 0.04 mm (3 SD) for the TrueBeam's (HDMLC and Millennium MLC) and for the Clinac (Millennium MLC), respectively.

#### 3.1.3 Sensitivity analysis

The sensitivity of the stakitt test is presented as the difference between the detected leaf position and gap width errors relative to the expected error magnitudes in Tables 2 and 3, respectively, for combinations HDMLC/Truebeam STx, Millennium120 MLC/Truebeam, and Millennium120 MLC/Clinac. Negative values indicate that the stakitt test under‐estimated errors whilst positive values indicate over‐estimation. The average difference (±1 SD) between nominal introduced error and measured error in leaf position across all error magnitudes for HDMLC/Truebeam STx was −0.135 ± 0.096 mm for the inner leaves. The same for Millennium120 MLC/Truebeam and Millennium120 MLC/Clinac were −0.027 ± 0.021 and −0.032 ± 0.018 mm, respectively. For gap width errors the average differences, again for inner leaves, were −0.048 ± 0.035 mm for HDMLC/Truebeam STx, −0.015 ± 0.015 mm for Millennium120 MLC/Truebeam, and −0.012 ± 0.013 for Millennium120 MLC/Clinac.

**TABLE 2:** Detected leaf position errors (± 1 SD) for the HDMLC on Truebeam Stx, Millennium MLC on TrueBeam, and Millennium MLC on Clinac.

	Measured leaf position deviation from expected (mm)		
Nominal induced error (mm)	TrueBeam HDMLC	TrueBeam Millennium	Clinac Millennium
−1.5	−0.23 ± 0.12	0.05 ± 0.02	0.05 ± 0.02
−1.0	−0.16 ± 0.09 (2.5 mm) −0.03 ± 0.02 (5 mm)	0.04 ± 0.02 (5 mm) 0.00 ± 0.02	0.04 ± 0.02 (5 mm) 0.01 ± 0.02 (10 mm)
−0.5	−0.09 ± 0.04	0.02 ± 0.01	0.02 ± 0.02
−0.1	−0.01 ± 0.01	0.00 ± 0.01	0.01 ± 0.01
0.1	−0.02 ± 0.01	0.00 ± 0.01	0.01 ± 0.02
0.5	−0.10 ± 0.05	0.02 ± 0.01	0.03 ± 0.03
1.0	−0.19 ± 0.08 (2.5 mm) −0.05 ± 0.02 (5 mm)	0.04 ± 0.01 (5 mm) 0.00 ± 0.01 (10 mm)	0.05 ± 0.03 (5 mm) −0.01 ± 0.02 (10 mm)
1.5	−0.28 ± 0.11	0.05 ± 0.01	0.06 ± 0.03

Numbers in brackets indicate leaf thicknesses where the same nominal induced error has been simulated for both inner and outer leaves.

**TABLE 3:** Detected leaf gap errors (± 1 SD) for the HDMLC on Truebeam Stx, Millennium MLC on TrueBeam, and Millennium MLC on Clinac.

	Measured gap deviation from expected (mm)		
Nominal induced error (mm)	TrueBeam HDMLC	TrueBeam Millennium	Clinac Millennium
−1.5	−0.07 ± 0.08	−0.03 ± 0.02	−0.03 ± 0.04
−1.0	−0.05 ± 0.06	−0.01 ± 0.02	−0.03 ± 0.03
−0.5	−0.03 ± 0.03	0.00 ± 0.02	−0.01 ± 0.02
−0.1	0.00 ± 0.01	0.01 ± 0.01	0.00 ± 0.02
0.1	−0.02 ± 0.01	−0.01 ± 0.01	0.00 ± 0.03
0.5	−0.04 ± 0.04	−0.02 ± 0.02	0.00 ± 0.03
1.0	−0.08 ± 0.06 (2.5 mm) −0.03 ± 0.02 (5 mm)	−0.03 ± 0.02 (5 mm) −0.02 ± 0.01 (10 mm)	0.00 ± 0.04 (5 mm) 0.00 ± 0.03 (10 mm)
1.5	−0.10 ± 0.08	−0.04 ± 0.02	−0.02 ± 0.03

Numbers in brackets indicate leaf thicknesses where the same nominal induced error has been simulated for both inner and outer leaves.

**FIGURE 4:**
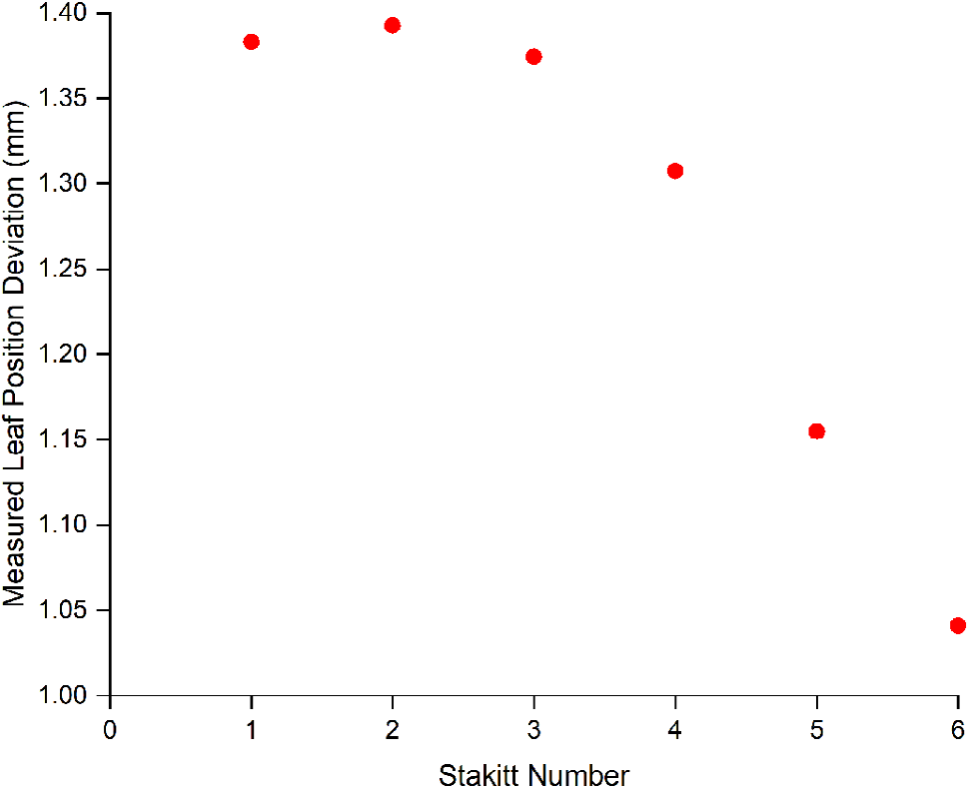
Example stakitt measured leaf position deviation for a 1.5 mm nominal error across the six stakitts.

The results of Table 2 (leaf position error) and Table 3 (MLC gap error) indicate that the stakitt method is sensitive to errors of clinically meaningful magnitudes with accuracy measured to within 0.1 mm. This is consistent between the Clinac and TrueBeam linacs and for the HD and Millennium MLC types. Of note in the results of Table 2 is that the accuracy of error detection decreases as the error magnitude increases for the HDMLC. The effect is not observed for the Millennium MLC or even on the wide outer leaves of the HDMLC and is just contained to the inner 2.5 mm leaves of the HDMLC. As per Table 3, the effect does not translate into the measured MLC gap, which has been reported as the clinically significant parameter.^10^, ^11^, ^13^, ^34^ Interrogating the data further revealed that the reduced accuracy for the small inner leaves for the HDMLC appeared to be dependent on the point along the leaf trajectory. This is demonstrated for a single leaf for the 1.5 mm nominal introduced error in Figure 4 and is representative of the other sensitivity experiment results.

#### 3.1.4 Comparison with picket fence

The stakitt MLC gap sensitivity data for the Clinac Millennium MLC was compared and contrasted against the previously published department picket fence test^22^ for the same experiment. Results are presented in Table 4.

**TABLE 4:** Gap width error detection (± 1 SD) comparison between stakitt and picket fence for the Clinac Millennium MLC.

Nominal induced error (mm)	Measured deviation from nominal (mm)	
	Stakitt	Picket Fence
0.1	0.00 ± 0.03	0.01 ± 0.02
0.5	0.00 ± 0.03	0.02 ± 0.01
1.5	−0.02 ± 0.03	0.00 ± 0.01

#### 3.1.5 Clinical use case

Mean results from the stakitt and backlash data are presented in Table 5.

**TABLE 5:** Clinical use case stakitt and Varian backlash test results.

	Deviation from nominal (mean ± 1 SD mm)		
	Bank A	Bank B	Gap
Stakitt			
G0	−0.42 ± 0.01	0.32 ± 0.02	−0.10 ± 0.01
G270	−0.49 ± 0.01	0.38 ± 0.01	−0.11 ± 0.00
G90	−0.22 ± 0.03	0.06 ± 0.04	−0.16 ± 0.00
Mean Difference (G90‐G270)	0.27 ± 0.04	− 0.32 ± 0.05	
Backlash			
G0	0.27 ± 0.04	0.28 ± 0.04	
G270	0.06 ± 0.05	0.05 ± 0.04	
G90	0.05 ± 0.04	0.05 ± 0.03	
Carriage	0.03 ± 0.00	0.03 ± 0.00	

The results of Table 5 show that the mean change in measured leaf deviation between G90 and G270 was 0.27 mm for Bank A in the direction which increases the gap and 0.32 mm for Bank B in the direction that decreases the gap. In terms of gap size, these results effectively cancel each other meaning that the gap size remain approximately constant, just offset in position. This is borne out in the measured mean gap errors between G90 and G270, which are measured to be ‐0.16 and ‐0.10 mm, respectively. The Varian backlash test is always performed at G0. At G90 and G270, the gravitational force overwhelms the backlash and hence backlash is measured to be negligible as demonstrated in Table 5. The carriage backlash were also measured to be negligible. As such, the mean change in stakitt results between G90 and G270 is compared to the backlash results at G0 and is found to agree within measurement uncertainty. This supports the hypothesis that the difference in measured MLC positions between G90 and G270 is almost exclusively due to backlash in the MLC leaves.

## 4 DISCUSSION

The design of the stakitt test pattern and analysis software addresses issues identified in the traditional picket fence test. The relatively wide stakitts (2 cm) avoids the penumbra on penumbra overlap associated with the picket fence 1 mm gap/overlap width. This allows for individual MLC leaf positions to be measured directly rather than the indirect method of measuring a picket position and picket width associated with the standard picket fence which make diagnosing which of the opposed MLC leaves is in error difficult. This also negates the need for a calibration step to determine the picket width from the full width half maximum. By spatially referencing to the measured CAX of the day, stakitt removes EPID panel positioning reproducibility error and allows for a measurement in absolute terms. By measuring along the MLC trajectory, determined directly from the image, collimator versus EPID panel rotation error is reduced, and by including a carriage shift in the test pattern, this aspect of the MLC positional performance is also assessed. With all of these improvements, the stakitt test is fundamentally superior to the traditional picket fence test for routine testing of MLC positioning.

The algorithm and repeatability results indicate that the algorithm is consistent and that measurements are repeatable to a magnitude smaller than the required tolerance of the test. The repeatability experiment includes repeatability of the MLC itself combined with the stakitt methodology repeatability. The inferior repeatability of the Clinac results compared to the TrueBeam results are considered likely due to slightly worse repeatability of the Clinac MLC positioning itself.

**FIGURE 5:**
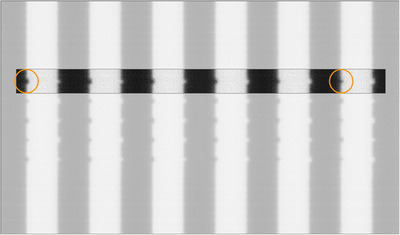
HDMLC 1 mm error stakitt image. The leaf with 1 mm error on the left hand side of the image is visibly wider than the same leaf on the right hand side of the image. The leaf presented is the same one as analyzed in Figure 4.

The sensitivity to introduced error results of Tables 2 and 3 suggest accuracy of the stakitt test to clinically acceptable levels even for the inner HDMLC leaves for which accuracy is reduced. The reduced error detection accuracy for the small inner leaves for the HDMLC was investigated and hypothesized to be due to an apparent radiological narrowing of the MLC leaves, which can be observed in Figure 5, which is a HDMLC stakitt image with a deliberate 1 mm error introduced in a single leaf. In Figure 5, there is an apparent narrowing of the error induced leaf, as it travels from left to right across the image. This also affects where the 50% point lies and hence the measured location of the leaf tip. The effect only becomes significant for the narrow 2.5 mm leaves.

**FIGURE 6:**
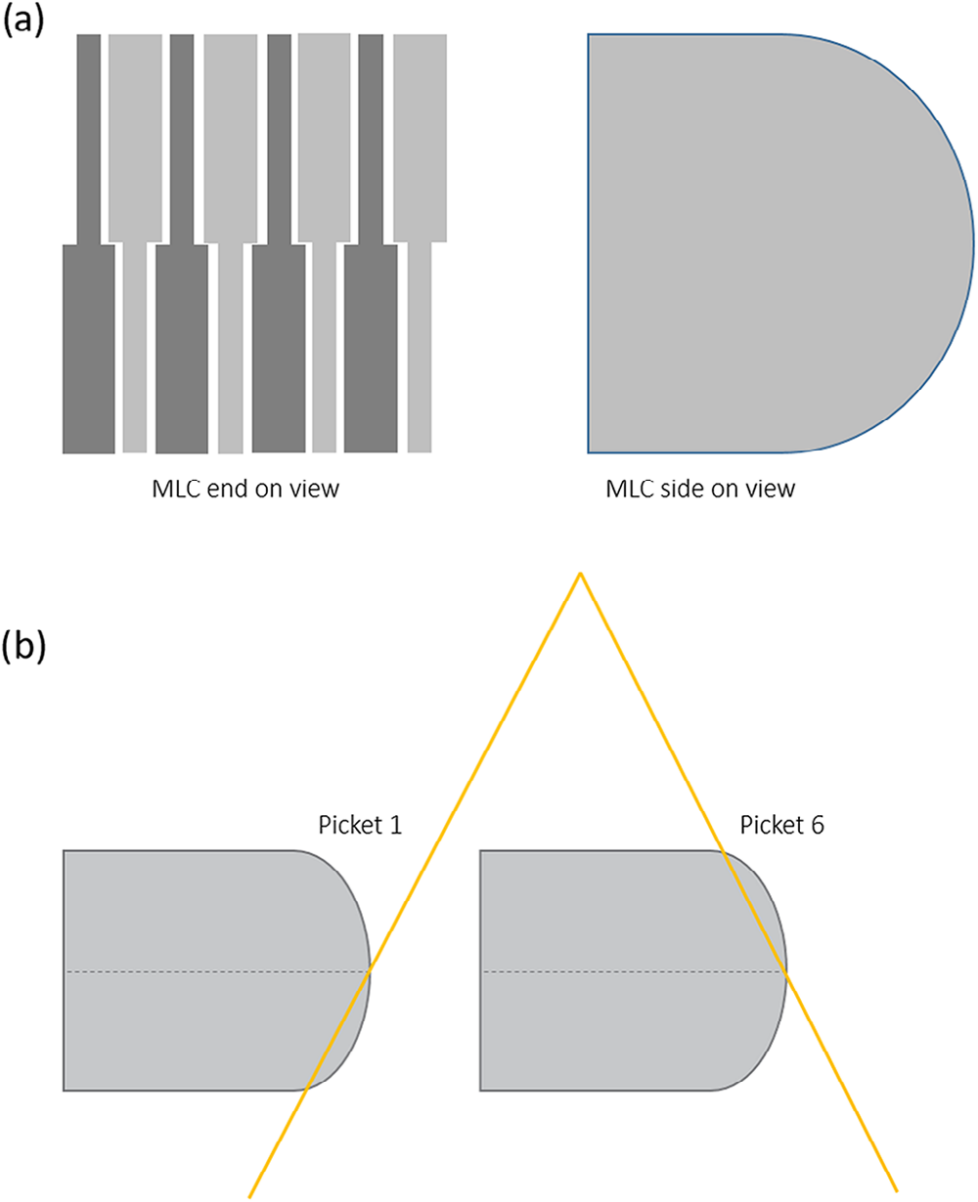
a) End‐on and side‐on views of the Varian MLC illustrating the different thickness section through the height if the MLC leaf as reported in Siebers et al.^35^ and Aitelcadi et al.^36^ b) Illustration that demonstrates that at on the open side of the MLC trajectory that the lower portion of the leaf collimates the beam whilst on the closed portion of the trajectory it is the top half that is collimating the beam.

The radiological narrowing was observed to occur for every second leaf with radiological widening observed for the alternate leaves. The phenomenon can be explained by the design of the Varian MLC leaves. Figure 6a provides an end‐on viewpoint of the Varian MLC, and as reported by Siebers et al.^35^ and Aitelcadie et al,.^36^ it can be noted that the tongue‐and‐groove arrangement results in leaves having a thick and a thin section through the thickness of the leaf, which alternate with leaves. As per Figure 6b as a leaf moves across the field, due to beam divergence, on one side of the field, it is the lower part of the leaf that predominantly defines the beam edge, while on the other side it is the upper part. Hence, dependent on whether the thick part of the leaf is collimating the beam or the thin section will determine the apparent thickness of the leaf when it is protruded from its neighbors. This also explains why adjacent leaves alternate between narrowing and widening. If the theory is correct, then the effect is real and to the authors’ knowledge has not been previously published as a consideration for quantitative MLC positional QA analysis. The effect is only present when individual leaves are protruding beyond their neighboring leaves.

The results of Figure 4 provide further insight into the radiological narrowing phenomenon.

As the stakitt number increases in Figure 4, the MLC leaf radiologically narrows as per Figure 5. As this occurs, the measured leaf deviation in Figure 4 appears to initially remain stable (stakitts 1, 2, and 3) before steadily decreasing (stakitts 4, 5, and 6) resulting in reduced measurement sensitivity. This behavior demonstrated in Figure 4 is representative of all the sensitivity experiment results. It is hypothesized that as the leaf radiologically narrows, it eventually gets so narrow that the profile along the leaf travel used to determine the 50% point and hence leaf tip position may not pass through the peak signifying the leaf tip. Theoretically, this would lead to the 50% point distance being shortened resulting in the reduced sensitivity observed in Figure 4. Figure 4 would indicate that there is a threshold for apparent leaf width whereby as width reduces the algorithm initially retains accuracy before reaching a narrowness beyond which the algorithm steadily loses accuracy.

The results of Table 4 show that both the picket fence and stakitt test detected the errors introduced accurately to the order of hundredths of a millimeter. This level is considered clinically excellent, however the value of the picket fence test results are limited as the gap width results require a calibration curve. This calibration step is problematic in the picket fence test as without a ground truth, assumptions need to be made about the accuracy of the actual MLC positions used in the calibration procedure. Also, this calibration step makes the picket fence sensitivity results (Table 4) mis‐leading as an image with intentional errors has been assessed by the test which has also been calibrated via a similar image with intentional errors meaning that actual errors may be masked in the analysis. The results of Table 4 indicate that the stakitt methodology can produce similar levels of accuracy without the need for the potentially flawed calibration procedure.

The clinical use case demonstrates the utility of stakitt in the clinical setting and provides insight into MLC positional behavior under gravity when backlash is present. In this regard, this complements the work of Barnes et al.^37^ in indicating that MLC positional variation with gantry angle is primarily due to backlash in the MLC, most likely caused by the t‐nut. This is further demonstrated in the presented clinical use case where differences in stakitt results between G90 and G270 were of the magnitude of the measured backlash via the in‐built Varian test. This finding calls into question the practicality of the MPPG 8.b. recommended action limit of ± 0.5 mm for MLC positioning accuracy at all cardinal gantry angles. Considering that backlash has been demonstrated to translate directly into MLC positioning error and that the current Varian tolerance on backlash is 0.4 mm,^36^ then a ± 0.5 mm action limit may be currently impractical. The Varian tolerance is not a hard tolerance and leaf backlash can be outside this tolerance without delivery being prevented. With such backlash present, the MLC would have to have no other source of positional error to be able to meet the proposed action limit. As such, linac vendors are called upon to improve the MLC t‐nut's such that the backlash tolerance can be reduced so that MPPG 8.b. recommendations can be practically met. In the interim, an action limit on MLC positional accuracy of ± 0.5 mm is suggested at G0 and the previous ± 1.0 mm action limit be retained for the other cardinal gantry angles.

The stakitt G0 results presented in Table 5 for the clinical use case more closely resemble the G270 results. This is expected. As shown in Figure 3, during a clockwise rotation, at G270, gravity is working to extend the leaves of Bank A and retract the leaves of Bank B. The magnitude of this influence reduces as the gantry is rotated toward 0°, but does not actually start to work in the opposing manner until after gantry has rotated past G0. Therefore, at G0, the effects of gravity can be expected to be consistent with the G270 result. However, the G0 results would be expected to resemble the G90 results if the measurements had followed an anti‐clockwise order.

The implementation of the stakitt test is similar to a traditional EPID‐based picket fence, as both require similar but different test plans to be created, both are acquired similarly with an integrated EPID image of the delivery, and both require custom analysis code of similar complexity. As such, the barrier to entry for stakitt compared to the traditional picket fence test is considered to be comparable and could be adopted by third‐party linac QA vendors for inclusion in their test suites. Stakitt provides a simple upgrade on current practices that has been demonstrated to be able to fulfill the MLC positional QA requirements recommended in MPPG 8.b.

## 5 CONCLUSION

An MLC positional test that is not susceptible to the known weaknesses of the picket fence test has been developed and evaluated and found to generally be highly accurate. The exception is with the HDMLC small leaves where accuracy was found to decrease, but still be within acceptable levels. The test has highlighted an apparent radiological variation in MLC leaf width along its trajectory that to the author's knowledge had not previously been presented in the literature as a consideration for quantitative MLC positional QA analysis. The accuracy levels achieved by the test allow for tightened MLC positional action limit of ± 0.5 mm recommended in AAPM MPPG 8.b. to be implemented. The test has demonstrated utility in the clinic for revealing MLC behavior and has highlighted that the MPPG 8.b. action limit of ± 0.5 mm may not be practical at non‐zero gantry angles. The stakitt test would appear to be an upgrade on the picket fence test that would be easy to implement.

## AUTHOR CONTRIBUTIONS

Michael Barnes (co‐primary author): Developed the original idea for the study, performed analysis, and interpretation of results and primarily wrote the manuscript.

Therese Standen (co‐primary author): Developed the stakitt test delivery plan and wrote the analysis code. Processed and analyzed the Stakitt data, contributed to the manuscript write up and provided scientific input.

Renee Blackmore: Worked on the clinical case study analysis and write up.

Peter Greer: Provided scientific input into the study and review of the draft manuscript.

## CONFLICT OF INTEREST STATEMENT

The authors acknowledge that they have no conflicts of interest relevant to this study.
